# Electronic Interactions in Coulombic Associated Photoactive Macrocycles to Chemically Modified MoS_2_ Nanosheets

**DOI:** 10.1002/chem.202404746

**Published:** 2025-02-19

**Authors:** Marina Tsigkou, Eleni Nikoli, Ioanna K. Sideri, Michalis Kardaras, Hiram Joazet Ojeda Galvan, Mildred Quintana, Nikos Tagmatarchis

**Affiliations:** ^1^ Theoretical and Physical Chemistry Institute National Hellenic Research Foundation 48 Vassileos Constantinou Avenue 11635 Athens Greece; ^2^ High Resolution Microscopy-CICSaB and Faculty of Science Universidad Autonóma de San Luis Potosi Av. Sierra Leona 550, Lomas de San Luis Potosi 78210 SLP Mexico

**Keywords:** MoS_2_, electrostatic association, Zn-phthalocyanine, Zn-porphyrin, boron dipyrromethene

## Abstract

Whilst functionalization of transition metal dichalcogenides (TMDs), and more specifically MoS_2_, has flourished the past decade, the accommodation of photoactive molecules on their lattice has unlocked the potentiality of this family of materials in a series of optoelectronic and energy related applications. The electronic communication between the chromophore and MoS_2_, in such systems, has been thoroughly studied, in cases where the grafting of the former on the latter is secured through covalent bonding. However, comparatively less attention has been drawn in cases where the chromophore is electrostatically anchored on MoS_2_, a means that potentially provides alternative ways of spatially accommodating the ligand on the TMD's extended environment, which directly affects the electronic communication between the two entities. In this work, we comparatively study the photophysical characteristics of three separate nanoensembles, where MoS_2_ is electrostatically hosting a Zn‐phthalocyanine, a Zn‐porphyrin, and a boron‐dipyrromethene, that complementary cover a wide range of visible absorption, via UV‐Vis and photoluminescence spectroscopy. The results highlight the strong interactions in the excited state across all chromophores, while the ground‐state interactions vary from chromophore to chromophore, indicating a distinct energy exchange dependent on the specific nanoensemble.

## Graphical abstract

Three different nanoensembles interfacing 2H‐MoS_2_ and Coulombic‐associated Zn‐metalated phthalocyanine, Zn‐metalated porphyrin and a boron dipyrromethene, are described and fully characterized. The three nanoensembles, complementary covering a wide visible absorbance range, are comparatively studied, with a focus on the electronic communication achieved between MoS_2_ and the respective photoactive molecules, in both the ground and the excited state.

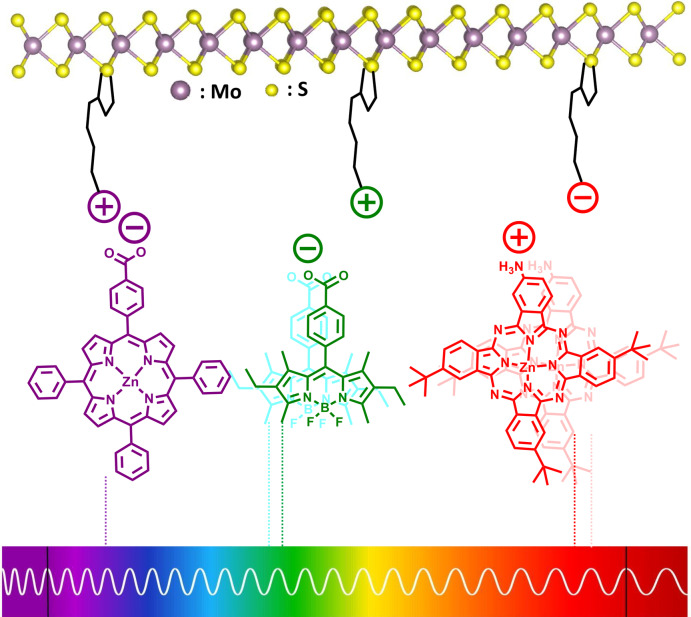

## Introduction

Two‐dimensional transition‐metal dichalcogenides (2D‐TMDs) have become a focal point in modern research due to their unique structure and remarkable physicochemical properties.[^1^, ^2^] Among 2D‐TMDs, 2H‐MoS₂ nanosheets stand out for their high accessibility and chemical stability, making them the most extensively studied material, in this category. Preparation of few‐layered MoS₂ can be realized by either top‐down synthesis methods or exfoliated techniques, which outweigh the former in the sense that allow precise control over key parameters, including the material's phase, lateral dimensions, and structural defect density.^[3]^ While exfoliation provides a straightforward approach to tuning 2H‐MoS₂ physical and structural characteristics, substantial progress has also been made in the chemical functionalization of 2D‐TMDs with organic molecules, providing an additional, reliable and targeted method for modulating the properties of these materials or even acquiring new ones.[^4^, ^5^] Towards the realization of the latter, when the organic molecules of choice are photoactive ones, the newly prepared systems exhibit interesting photoluminescence and redox properties, which apply in a series of energy‐conversion processes, featured in optoelectronic and light‐harvesting applications.^[6]^ Hence, extensive research has been centered on MoS₂‐based nanohybrids bearing photoactive species, that are grafted on the nanosheets via covalent bonding, whereas particular emphasis has been devoted on charge transfer, charge carrier doping, and charge injection processes, within the novel systems.[^7^, ^8^, ^9^, ^10^]

Regarding covalently functionalized MoS₂ nanosheets, based on the specific photoactive molecule – such as carbon dots (CDs), phthalocyanines, porphyrins and perylene diimides (PDIs) – a range of different photophysical responses has been registered. Diverse electronic behaviors of MoS_2_ could be exhibited through different functionalization. Upon functionalization with CDs, MoS_2_ acts as an electron donor and/or energy acceptor.^[7]^ In the presence of phthalocyanines, MoS_2_ functions as a bidirectional electron acceptor,^[8]^ whereas interaction with porphyrins enables it to serve as an energy reflector.^[9]^ When combined with an electron‐withdrawing PDI derivative, MoS_2_ reveals its electron‐donating ability.^[10]^ In addition, a couple of studies regarding the association of MoS_2_ with boron dipyrromethene (BODIPY) dyes have been explored, albeit their focus was not related to the photoinduced electronic properties of the hybrid systems.[^11^, ^12^]

However, comparatively less attention has been focused on respective systems, where the selected chromophore moiety is non‐covalently interacting with the TMD nanomaterial. Non‐covalent association of MoS₂ with porphyrins^[13]^ and various phthalocyanines – both metal‐centered (e. g., magnesium (MgPc),^[[14]]^ copper (CuPc), cobalt (CoPc), zinc (ZnPc)) and metal‐free (H₂Pc)^[[15]]^ – has also been explored, albeit to a lesser extent. In these supramolecular ensembles, MoS₂ can act as either an electron donor or acceptor, depending on the HOMO‐LUMO energy levels of the interacting organic molecule.[^16^, ^17^] Notably, in most of these studies, the modified MoS₂ nanosheets are prepared by simple physical mixing of the chromophore with the exfoliated MoS₂ nanosheets.[^13^, ^14^, ^15^, ^16^, ^17^] Although this methodology takes advantage of the Van der Waals interactions between monolayer MoS_2_ and the chromophore molecules, leveraging the S‐π interactions between them,[^13^, ^14^, ^15^, ^16^, ^17^] those are weak, do not provide control over the degree of functionalization, therefore do not guarantee reproducibility, consistency or durability, when aiming in the fabrication of devices.

An alternate way to overcome this setback and at the same time heal the defects of MoS_2_ lattice, is the covalent functionalization of MoS_2_ with organic moieties through defect engineering and the subsequent electrostatic association with the chromophore of choice.[^18^, ^19^, ^20^, ^21^] The covalent attachment of organic moieties to MoS₂ significantly enhances both its dispersibility^[22]^ and colloidal stability,^[23]^ with the organic chains serving as an effective physical barrier that prevents aggregate formation, through steric hindrance, and reduces Van der Waals interactions between MoS₂ nanosheets. In this direction, the use of sulfur containing organic molecules is broadly utilized aiming to modify MoS_2_ by healing sulfur vacancies already existing in its lattice. Although the covalent character of the interaction between thiols and MoS_2_ in the resulting modified material has been questioned,^[24]^ the use of alternate sulfur sources, like in our case the utilization of 1,2‐dithiolane derivatives, has been extensively used and proved to be very robust.^[23]^  Having a rigid first functionalization step secure, a subsequent electrostatic second functionalization step is possible, securing the desired stability and reliability of the system, while also allowing the study of the electronic communication between the MoS_2_ nanosheet and the photoactive counterpart. Following this approach, overall offers a more efficient way to tailor the material‘s properties, facilitates a better investigation of its behavior in aqueous environments, and provides a more straightforward approach for realizing electronic communication in such systems.

In our previously reported study,^[18]^ positively charged ammonium‐modified 2H‐MoS_2_ formed a stable electrostatic association with a porphyrin bearing a carboxylate moiety, while the transduction of energy from the photoexcited porphyrin to MoS₂ was confirmed. Notably, the porphyrin employed lacked a metal center, highlighting an area that warrants further exploration, especially considering the capabilities that are unlocked, application‐wise in the presence of a non‐precious metal in such systems. Analogous results were showcased in the case of electrostatically attached CDs^[19]^ and polythiophenes^[20]^ on MoS_2_, where electron/energy transfer from their singlet excited state to the TMD was observed as the deactivation pathway.

Building on numerous studies of covalent grafted photoactive components on TMDs, the aim of the present study is to explore electronic communication in analogous systems, characterized by non‐covalent grafting means. Here, we present a comparative study of 2H‐MoS_2_ functionalized with three different photoactive molecules, electrostatically associated to MoS_2_. In the present work, surface modification of MoS₂ is achieved through the covalent attachment of specially designed organic chains onto the 2D nanosheets, resulting in easily protonated/ deprotonated 2H‐MoS₂ nanosheets. The Coulombic interactions that may occur between the ionized nanosheets and selected counter‐ionized photoactive species, allow for an in‐depth study of the electronic communications taking place between MoS_2_ and the photoactive molecules by electrostatic wiring. The selected chromophores include a zinc phthalocyanine (ZnPc) modified to bear an amino group, as well as a zinc porphyrin (ZnP) and a boron dipyrromethene (BODIPY) derivative, bearing a carboxylic acid group. Phthalocyanines and porphyrins share structural similarities, both containing 18 π‐electron systems, and exhibit notable electronic properties, such as high molar absorption coefficients and well‐studied photoinduced energy and electron transfer properties.[^25^, ^26^] Furthermore, their properties can be tailored by the incorporation of various peripheral substituents and/ or metal centers.^[27]^ Additionally, they demonstrate exceptional chemical and thermal stability, high photostability, and potent photosensitizing properties in the visible and near‐infrared region of the electromagnetic spectrum.^[28]^ On the other hand, BODIPY is another class of structural analogue to porphyrin, often regarded as a versatile platform due to its neutral charge, well‐defined absorption and emission profile, and high fluorescence efficiency.[^29^, ^30^] The structure of BODIPY is highly amenable to modification, allowing even minor adjustments to significantly alter properties, such as absorption and emission wavelengths or hydrophilicity, thereby enhancing their applicability as light harvesting species.[^31^, ^32^] Furthermore, the three organic dyes were carefully selected as they complementary cover a broad absorption range in the whole visible region. Overall, this work presents a comparative study of the interactions between the aforementioned organic photoactive units and chemically modified 2H‐MoS₂ nanosheets, with a focus on the electronic interactions taking place between the counterparts, in the ground and excited state, that are associated in a Coulombic, non‐covalent manner.

## Results and Discussion

**Scheme 1:**
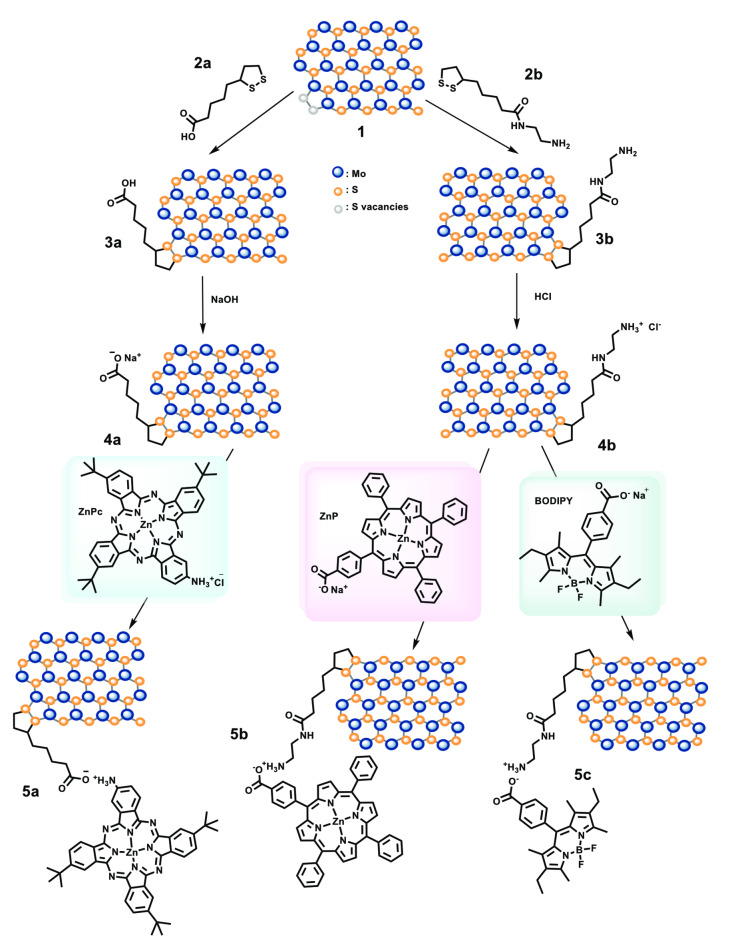
Illustrative modification of exfoliated 2H‐MoS_2_ nanosheets **1** through reaction with 1,2‐dithiolanes **2 a** and **2 b**, followed by deprotonation/ protonation, respectively, to yield negatively charged MoS_2_‐based material **4 a** and positively charged MoS_2_‐based material **4 b**, respectively. Ionic exchange with **ZnPc**, **ZnP** and **BODIPY** derivatives results in the formation of MoS_2_‐based nanoensembles **5 a**, **5 b**, and **5 c** via electrostatic interactions.

Preparation of few‐layered 2H‐MoS_2_ nanosheets **1** was accomplished via liquid phase exfoliation of bulk MoS_2_ in N‐methyl‐2‐pyrrolidone (NMP), adhering to a previously established protocol.[^3^, ^33^] UV‐Vis spectroscopy (Figure S1) confirmed the successful exfoliation, as evidenced by a shift in the exciton peak from 700 nm to 683 nm, indicating an average amount of 15 layers (see Supporting Information). The exfoliated 2H‐MoS_2_ nanosheets **1**, served, then, as the substrate to stepwise accommodate the desired chromophores via Coulombic interactions. In order to do so, initial covalent functionalization took place, employing 1,2‐dithiolane derivatives to passivate the existing S vacancies located mainly at the edges of the few layered 2H‐MoS_2_.[^23^, ^34^] Scheme 1 depicts the preparation protocol followed. Functionalization took place in two different directions, to afford both carboxylic‐acid‐terminated 2H‐MoS_2_ and amino‐terminated 2H‐MoS_2_, that would allow Coulombic interactions to take place in a following step. Hence, exfoliated 2H‐MoS_2_ nanosheets **1** were treated with α‐lipoic acid **2 a** and N‐(2‐aminoethyl)‐S‐(1,2‐dithiolan‐3‐yl)pentanamide **2 b**, yielding MoS_2_‐based materials **3 a** and **3 b**, respectively (Scheme 1). The synthesis of derivative **2 b** took place following an already existing procedure.^[35]^

**Figure 1:**
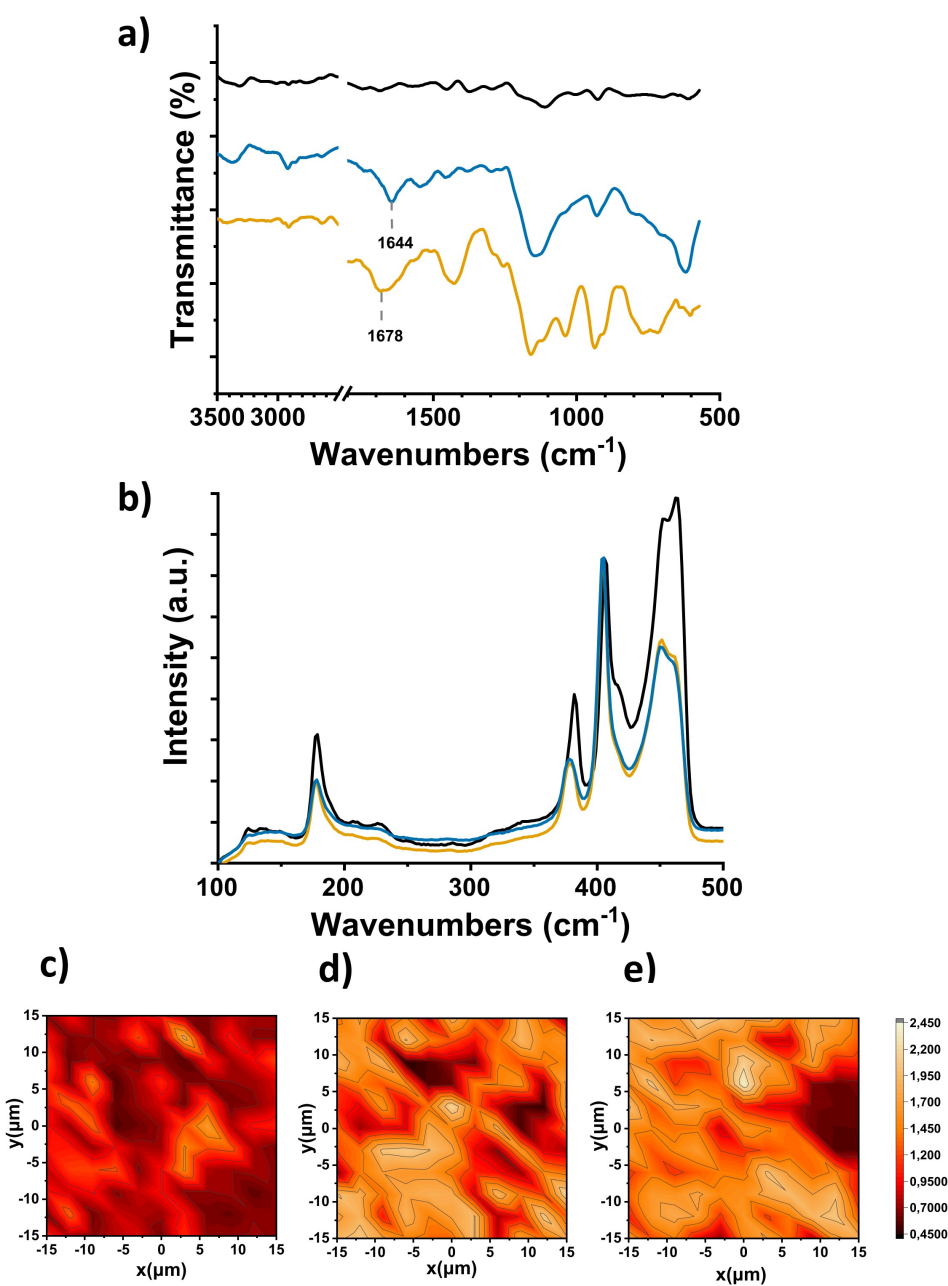
**a**) FT‐IR spectra, **b**) Raman spectra (633 nm) normalized at the A_1g_ mode of exfoliated MoS_2_ nanosheets **1** (black), modified materials **3 a** (orange) and **3 b** (blue). Raman spectral mapping (633 nm) of the Ι_A1g_/Ι_2LA(Μ)_ intensity ratio of a 15 μm x 15 μm area for **c**) exfoliated MoS_2_ nanosheets **1**, and, **d**, **e**) modified materials **3 a** and **3 b**, respectively.

In order to validate the successful preparation of **3 a** and **3 b**, complementary spectroscopic and thermal characterization was performed. In the FT‐IR spectra of both **3 a** and **3 b**, shown in Figure 1a, bands in the range of 2910–2920 cm^−1^, indicative of C−H stretching vibrations, were observed, along with characteristic bands at 1678 cm^−1^ and 1644 cm^−1^ corresponding to carbonyl stretching vibrations of the carboxylic acid for **3 a** and the amide moiety for **3 b**, respectively. In Figure S2 and Figure S3, the IR spectra of each modified material **3 a** and **3 b** are depicted, in comparison with their respective organic analogues **2 a** and **2 b**, respectively, confirming the attachment of each organic chain onto MoS_2_.

Raman spectroscopy (633 nm) was employed to provide further insight about the materials’ structure and the defects’ healing due to incorporation of **2 a** and **2 b**. The characteristic Raman spectral bands for 2H‐MoS_2_ (**1**) were evident (Figure 1b), namely the A_1g_‐LA(M) (180 cm^−1^), the in‐plane E^1^_2g_ (382 cm^−1^), the out of plane A_1g_ (405 cm^−1^) and the defect‐induced 2LA(M) (450 cm^−1^).^[36]^ Furthermore, the absence of the *J*_*1‐3*_ phonon modes, associated with the metallic polytype of MoS_2_, confirms the semiconducting character of exfoliated MoS_2_ nanosheets.^[37]^ These characteristic bands of 2H‐MoS_2_ nanosheets **1** are also evident in the Raman spectra of modified materials **3 a** and **3 b**, shown in Figure 1b, confirming the preservation of the semiconducting character of MoS_2_ upon functionalization. It is worth mentioning, however, that for normalized spectra at the A_1g_ mode (405 cm^−1^), the intensity of the 2LA(M) band at 450 cm^−1^ of **3 a** and **3 b** is decreased, compared to the one corresponding to exfoliated 2H‐MoS_2_ (**1**). The decrease in the intensity of the 2LA(M) band is well explained by considering that lattice S‐defects in exfoliated MoS_2_ were passivated upon functionalization and incorporation of the 1,2‐dithiolane derivatives **2 a** and **2 b**.^[38]^ Furthermore, 2D Raman spectral mapping provides a more comprehensive view of this intensity decrease of the 2LA(M) mode, as it collects spectral information over a broad area of the material, enabling the assessment of spatial consistency across the material. The intensity ratio I_A1g_/I_2LA(M)_ was mapped over a 15 μm x 15 μm area, for each modified material **3 a** and **3 b**, shown in Figure 1c **‐e**, with average values of ~1.35 for **3 a** and ~1.39 for **3 b**, respectively, compared to ~0.85 for exfoliated MoS_2_ nanosheets **1**, constituting strong evidence of the defect engineering process via the 1,2‐dithiolane addition.

**Figure 2:**
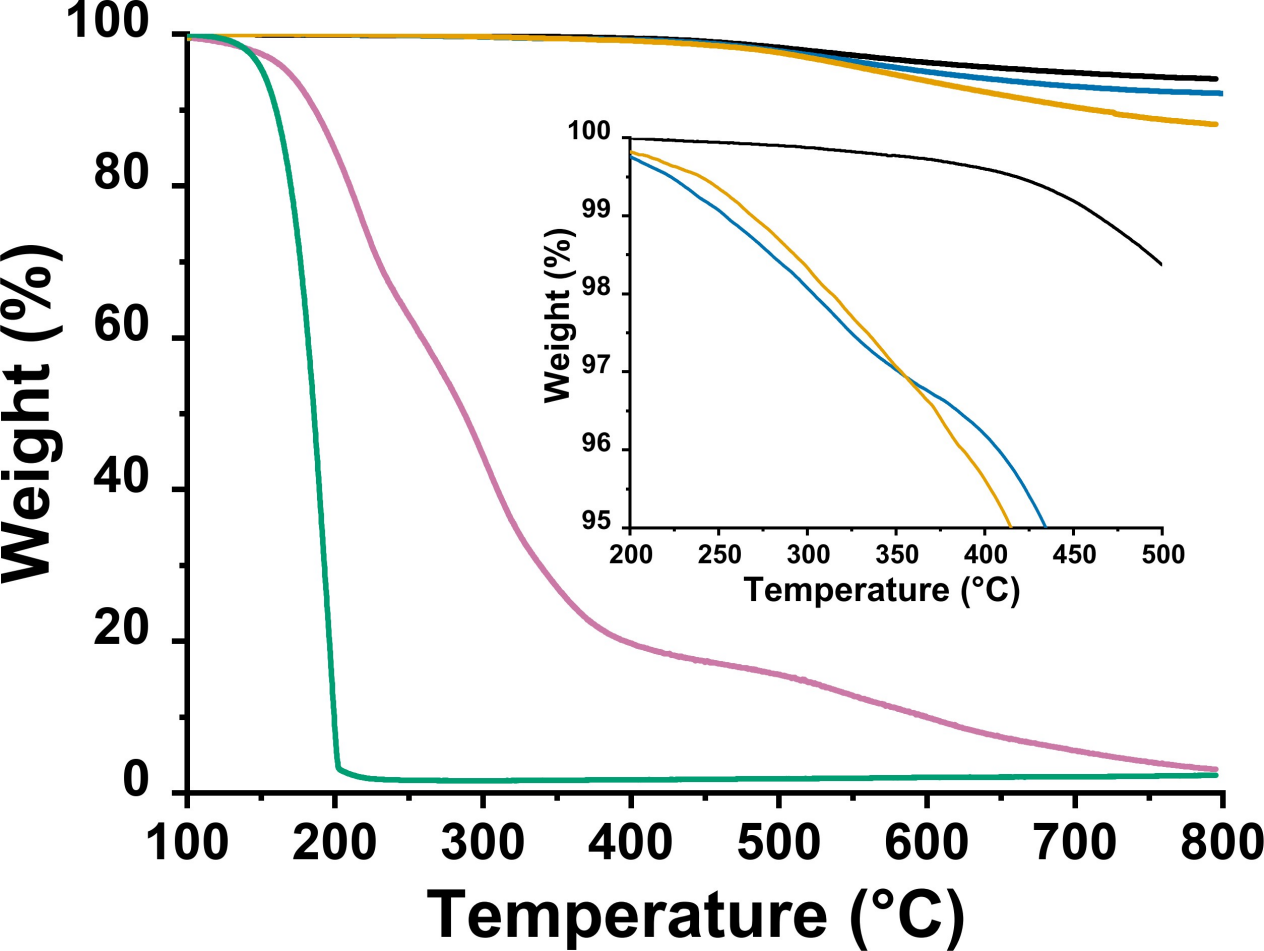
Thermographs of MoS_2_‐based modified materials **3 a** (orange) and **3 b** (blue), compared to exfoliated MoS_2_ nanosheets **1** (black), and 1,2‐dithiolane derivatives **2 a** (green) and **2 b** (purple).

Next, thermogravimetric analysis (TGA) of **3 a** and **3 b**, under nitrogen atmosphere, revealed the degree of MoS_2_ modification. Exfoliated 2H‐MoS_2_ nanosheets **1** exhibits thermal stability up to 450 °C, after which temperature the decomposition of the material starts at sulfur defected sites, as indicated by the increase of the weight loss (Figure 2). However, the thermographs related to the modified materials **3 a** and **3 b** show a greater weight loss percentage, at 400 °C, due to the decomposition of the incorporated organic addend onto MoS_2_, which quantitively corresponds to a weight loss of 4.2 % and 3.5 %, respectively. These results suggest that the degree of functionalization is approximately 1 organic moiety per 170 and 140 MoS_2_ units for **3 a** and **3 b**, respectively. The equation used for these calculations is provided in the Supporting Information section.

Envisioning the electrostatic association of the photoactive species **ZnPc**, **ZnP** and **BODIPY** with modified MoS_2_ nanosheets **3 a** and **3 b**, electric charges had to be introduced to the organic moieties covering the sulfur‐vacancies. Thus, the MoS_2_‐based modified material **3 a**, bearing a terminal –COOH unit as part of the addend, was treated with aqueous sodium hydroxide (0.5 M), to incorporate negatively charged carboxylate species onto MoS_2_ nanosheets **4 a**, as shown in Scheme 1. On the other hand, the MoS_2_‐based modified material **3 b**, bearing a terminal –NH_2_ unit as part of the addend, was treated with aqueous hydrochloric acid (0.01 M), to incorporate positively charged ammonium species onto MoS_2_ nanosheets **4 b**. In parallel, **ZnPc**, **ZnP** and **BODIPY** derivatives were acid‐ and base‐treated to yield their ionized form, able to electrostatically interact with our counter‐ionized MoS_2_‐based modified materials **4 a** and **4 b**.

**Figure 3:**
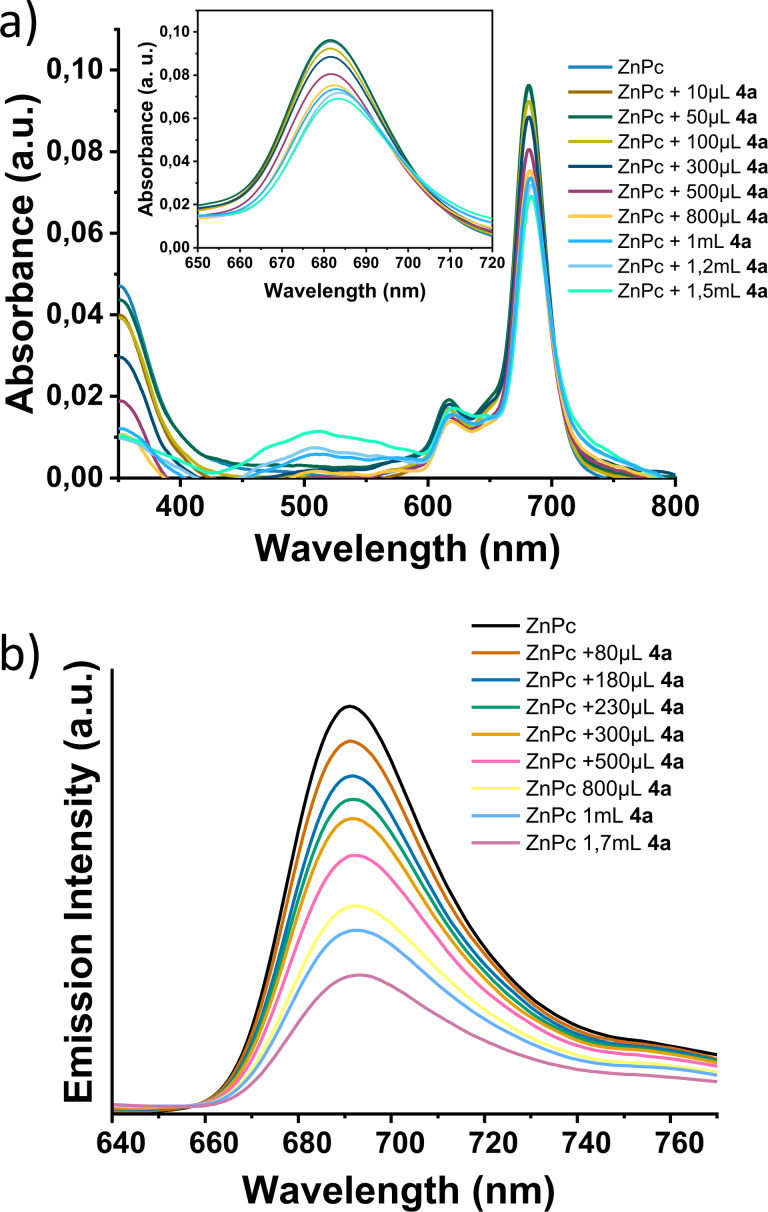
**a**) Absorption spectra of **ZnPc** upon sequential additions of **4 a** in DMF, after subtraction of the MoS_2_ absorption background. Inset: magnified view of the spectral region between 650 and 720 nm. **b**) Emission spectra of **ZnPc** upon incremental additions of **4 a**, in DMF (*λ_exc_* 615 nm).

In order to monitor the Coulombic association of the ionized **ZnPc**, **ZnP** and **BODIPY** derivatives, and the MoS_2_‐based materials **4 a** and **4 b**, titration assays were conducted. This method involved the incremental additions of a dispersion of a charged, modified MoS₂‐based material **4 a** or **4 b** to a solution of the selected photoactive species bearing an oppositely charged functional group **ZnPc**, or **ZnP**, and **BODIPY**. Throughout the titration assay, the concentration of each chromophore was maintained at a constant value of 10^−6^ M, while the amount of modified MoS₂ in the mixture was progressively increased, with dimethylformamide (DMF) serving as the solvent. This procedure is reported in further detail in the Supporting Information section. Each addition was followed by UV‐Vis and photoluminescence (PL) spectroscopy measurements. The acquired absorption spectra of **ZnPc** upon sequential additions of **4 a**, after subtraction of the MoS_2_ absorption background are depicted in Figure 3a, while in Figure 3b the corresponding PL spectra of **ZnPc** after each addition of **4 a** are shown. In parallel, UV‐Vis absorption spectra of **ZnPc** as acquired during the titration procedure in order to showcase the increasing MoS_2_ concentration as well as its contributing intrinsic absorption, are also shown in Figure S4. Figures S5 and S6, depict the UV‐Vis absorption spectra of **ZnP** during the titration with ‐and without, respectively‐, the subtraction of MoS_2_ absorption background. The corresponding UV‐Vis spectra for **BODIPY** are presented as Figures S7 and S8. Finally, the PL spectroscopy measurements of **ZnP** and **BODIPY**, after sequential additions of **4 b**, are shown as Figures S9 and S10, respectively.

In regards to the UV‐Vis spectra acquired for the titration of **ZnPc**, the Q band with its specific splitting in two maxima at 630 nm (π‐π* transition) and 681 nm (excitonic transition),^[39]^ is evident. It is important to note that the latter exhibits 3 nm red shift upon incremental additions of **4 a**, as shown in Figure 3a. To illustrate further, the absorption band of pure **ZnPc** at 681 nm, shifts to 684 nm after the formation of nanoensemble **5 a**. This shift leads to the formation of an isosbestic point at 703 nm, evident in the subtracted spectra (Figure 3a), and possibly indicating the association of the two components within nanoensemble **5 a**. Specifically, it suggests the development of electronic interactions between the two species in the ground state, resulting in a narrowing of the ZnPc bandgap. It is interesting that the absorption spectroscopy study of **BODIPY** titration towards the formation of **5 c** reveals an analogous pattern. To begin with, **BODIPY** absorption band appears centered at 524 nm, with a visible shoulder at around 490 nm, while upon addition of **4 b**, and consequently upon the formation of **5 c**, a 3 nm blue shift is observed, as far as the band centered at 521 nm is concerned, that is the maximum absorbance of the system (Figure S7). This is also particularly evident in the magnified spectral region of 500 to 550 nm, as depicted in the inset of Figure S7. In this case, the interaction between **BODIPY** and MoS₂ nanosheets in the ground state influences the HOMO (and/or LUMO) energy levels of **BODIPY**, in a different manner than **ZnPc** does to those of **5 a** nanoensemble. Figures S4 and S8 depict the absorption spectra recorded during the titration procedure of **ZnPc** and **BODIPY**, respectively, without subtraction of the MoS_2_ absorbance background.

On the contrary, regarding **ZnP** and **5 b**, the characteristic Soret absorption band of the porphyrin ring, which signifies the absorption maximum, is evident and centered at 426 nm, through the entire titration process, meaning that no shift is observed upon the incremental addition of MoS₂ (Figure S5). Likewise, the characteristic Q bands of **ZnP**, centered at 477 and 532 nm, appear to be equally unaffected.^[40]^ Figure S6 depicts the absorption spectra of the titration mixture of **ZnP** after each addition of modified MoS_2_ **4 b**, while in Figure S5, the representative spectra depicted were recorded with parallel subtraction of the modified 2H‐MoS_2_ absorption background, to showcase that the optical concentration of **ZnP** remains stable throughout the entire titration procedure. Upon the incremental addition of **4 b**, the resulting absorption spectra show the characteristic bands of the semiconducting phase of 2H‐MoS₂. Specifically, the excitonic A and B bands at 683 nm and 632 nm, corresponding to the direct transition from the spin‐orbit split valence band maximum to the conduction band minimum at the K point of the Brillouin zone and also the broad absorption bands C, centered at 470 nm, and D, centered at 400 nm, correspond to electronic transitions across the bandgap from the valence band maximum to the conduction band minimum at the M point of the Brillouin zone.^[41]^ In stark contrast with **ZnPc** and **BODIPY**, in the case of **ZnP** and the formation of nanoensemble **5 b**, no electronic interaction appears to develop between **ZnP** and MoS_2_ at the ground state. This is in agreement with our previous work in an analogous system, where a non‐metalated porphyrin was used instead.^[18]^ Notably, the introduction of Zn metal center in this work, red‐shifts the Soret absorption maximum about 5 nm as expected, but does not affect the way the two entities (MoS_2_ and porphyrin) interact in the ground state. Nonetheless, the introduction of the metal opens new avenues both structure‐wise and application‐wise.

In UV‐Vis spectroscopic assays, nanoensembles **5 a** and **5 c** exhibit a 3 nm red and blue shift, respectively, compared to the parent **ZnPc** and **BODIPY**. Additionally, both systems show strong, gradual quenching of emission intensity upon excitation at each chromophore's absorption maximum, as the MoS_2_ concentration increases (Figure 3b and Figure S10). As expected, this is also the case for the **ZnP** modified analogue in **5 b**. In detail, upon excitation of the chromophore molecules ‐ at 615 nm for **ZnPc**, 425 nm for **ZnP**, and 490 nm for **BODIPY** ‐ the emission intensity gradually decreases as the concentration of MoS₂‐modified materials **4 a** and **4 b** increases in each case (Figure 3b, Figure S9, and Figure S10, respectively). This trend suggests strong electronic interactions within nanoensembles **5 a**, **5 b**, and **5 c**, respectively. Quantitatively, in **5 a**, a 78 % quenching of the emission intensity of **ZnPc** is observed, until the gradual decrease reaches plateau, while the respective values for **5 b** and **5 c** are 70 % and 48 % quenched, with respect to the emission intensity of **ZnP** and **BODIPY**, respectively. It is important to note though that these values are not directly comparable, since each system requires different amount of modified MoS_2_ equivalent to reach plateau in the specific set up of the present study. More specifically, in the photoluminescence (PL) spectrum of **ZnP** (Figure S9), two characteristic emission bands, centered at 605 nm and 658 nm, are registered, while in the PL spectrum of **ZnPc** one characteristic band exists at 689 nm, with a 3 nm red shift for the so‐formed nanoensemble (**5 a**), as shown in Figure 3b. In parallel, **BODIPY** characteristic emission band, centered at 536 nm, is blue‐shifted by 3 nm, upon formation of nanoensemble **5 c**, as shown in Figure S10. With incremental addition of **4 a** and **4 b** to the titration mixture, the intensity of each band gradually decreases, until reaching a plateau. This trend confirms the successful formation of nanoensembles **5 a**, **5 b**, and **5 c**. The observed shifts, which are in total agreement with the respective absorption shifts, possibly indicate an alteration of the energy levels of the chromophores due to their interaction with the MoS_2_ nanosheets, leading to a widening of the **BODIPY** HOMO‐LUMO gap and a corresponding narrowing that of **ZnPc**. Also, the strong quenching effect registered, possibly indicates a deactivation pathway within each system via a charge or energy transfer mechanism. It is noteworthy that according to a previous study, where **ZnPc** was covalently attached on the edges of MoS_2_ nanosheets, the strong quenching effect registered for the so‐formed hybrid in comparison with intact **ZnPc**, was not accompanied by any shift neither in the UV‐Vis, nor in the PL studies. However, in a series of physical mixtures of **ZnPc** and MoS_2_ in varying ratios, a clear blue shift was observed in the PL emission of **ZnPc** after successive attachment of MoS_2_ due to photoinduced electron transfer from **ZnPc** to MoS_2_.^[42]^ Therefore, the red shift observed in this case, in both UV‐Vis and PL spectra, constitutes also a direct proof of the effect that the Coulombic association ‐as a means of interaction‐ has on the electronic communication between the two entities, that differs in covalently and non‐covalently bonded analogous systems.

**Figure 4:**
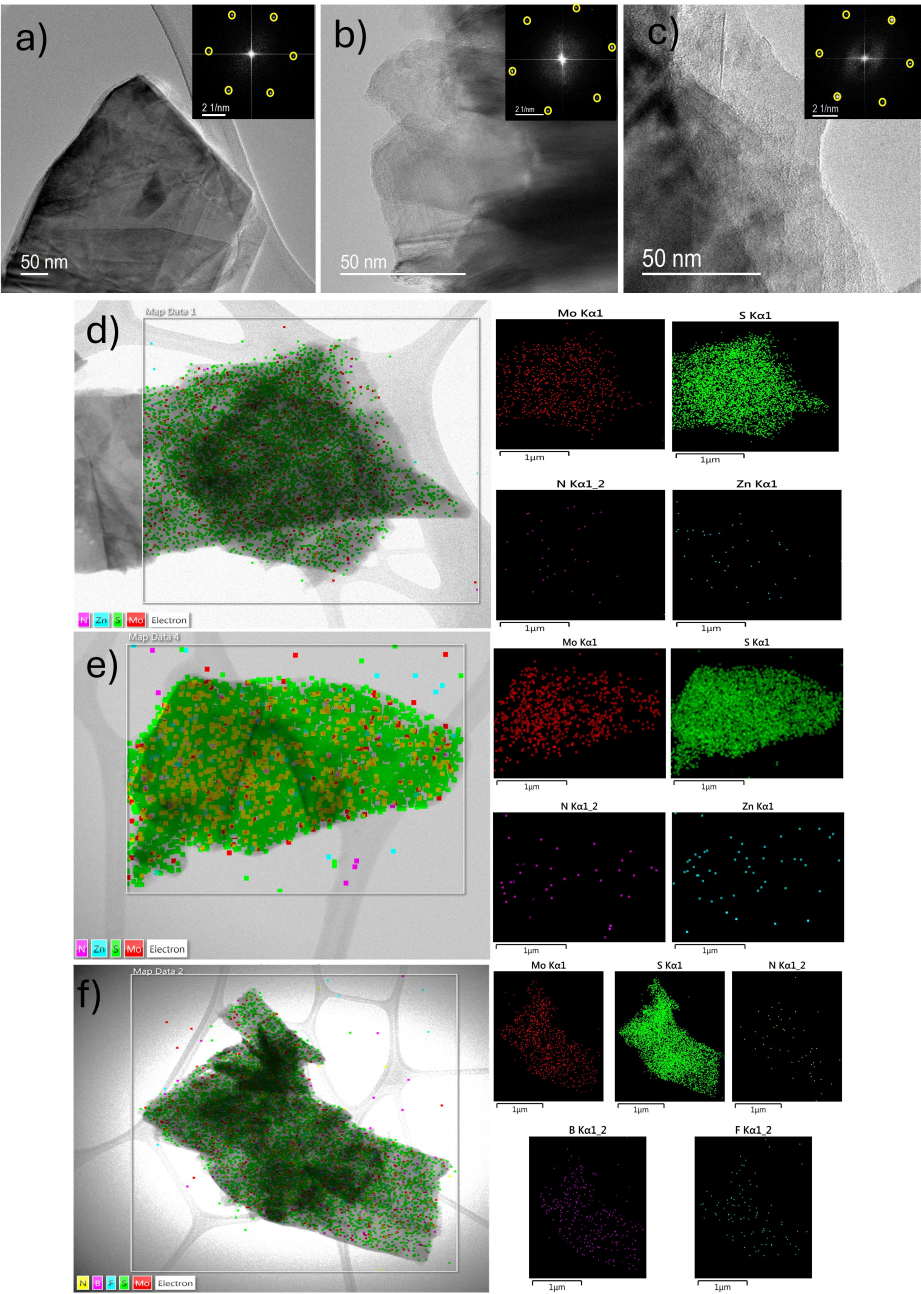
TEM imaging and EDS characterization of MoS₂ nanosheets electrostatically associated metal and metalloid macrocycles. **a**), **b**), and **c**) TEM images of MoS‐based **ZnP**, **ZnPc**, and **BODIPY** nanoensembles, respectively, showing the preserved layered structure of MoS₂ nanosheets. Insets: Fast Fourier transforms (FFTs) confirming the hexagonal symmetry characteristic of 2H‐MoS₂. EDS mapping of **d**) **MoS_2_/ZnP** nanoensemble **5 b**, displaying Mo, S, N, and Zn elements, **e**) **MoS_2_/ZnPc** nanoensemble **5 a**, revealing Mo, S, N, and Zn across the material, and **f**) **MoS_2_/BODIPY** nanoensemble **5 c**, displaying Mo, S, B, F, and N elements.

Transmission electron microscopy (TEM) imaging provided insights into the morphological and structural characteristics of the functionalized 2H‐MoS₂ nanosheets and their **ZnPc**, **ZnP**, and **BODIPY** nanoensembles **5 a**, **5 b** and **5 c**, respectively. TEM micrographs shown in Figures 4a, b, and c, for electrostatically associated MoS_2_ with **ZnP**, **ZnPc**, and **BODIPY** derivatives, respectively, reveal the exfoliated structure of the MoS₂ nanosheets, retaining their few‐layered architecture and hexagonal symmetry, as confirmed by the inset fast Fourier transforms (FFT). The interplanar spacing of the (100) plane was measured to be 0.27 nm, which aligns with reported values in the literature.^[43]^

Energy dispersive X‐ray spectroscopy (EDS) mapping, in Figures 4d, **e**, and **f**, for MoS_2_‐based **ZnP**, **ZnPc**, and **BODIPY** nanoensembles respectively, firstly reveals the presence of Mo and S as the support of MoS₂. At the same time, elements specific to the chromophores, such as Zn and N for **5 a** and **5 b**, and B, F, and N for **5 c**, distributed across the material‘s surface, demonstrating and verifying the effectiveness of the covalent functionalization allowing the Coulombic association of the chromophores.

To further assess the successful association between the photoactive species and exfoliated MoS₂ and spatially reenact the Coulombic association that is formed in wet media during the UV‐Vis and PL titration procedures already described, dynamic light scattering (DLS) measurements were conducted. In contrast to the TEM measurements, where the samples are imaged in powder form, DLS assays allow the observation of the formed nanoensembles in dispersion, where the size distribution measured is directly impacted by the potential formation of nanoaggregates. Hence, the average hydrodynamic radius (R_h_) of exfoliated MoS₂ was determined to be 235 nm. In contrast, the R_h_ values of the nanoensembles **5 a**, **5 b**, and **5 c** were found to be significantly larger, measuring 320 nm, 316 nm, and 350 nm, respectively (Figure S11). These results are consistent with expectations, as the photoactive species interfacing with MoS_2_ nanosheets are electrostatically adhered to the MoS₂ lattice, thereby increasing the overall hydrodynamic radius.

**Figure 5:**
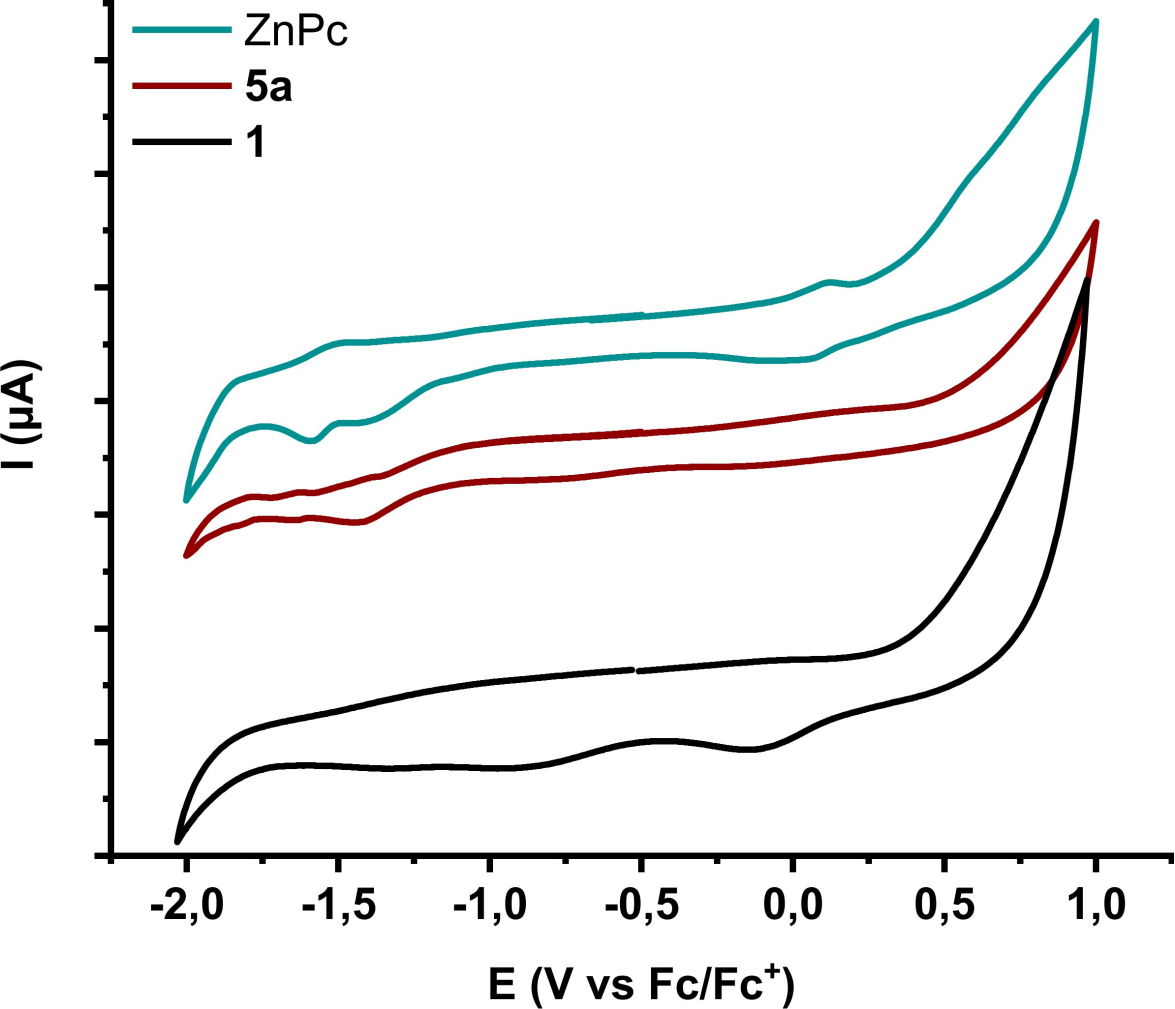
Cyclic voltammogram of exfoliated MoS_2_ (**1**), **ZnPc** and nanoensemble **5 a**, in dry DMF and nitrogen saturated 0.1 M TBAPF_6_ as electrolyte.

In order to gain further insights regarding the acquired chemical characteristics of the newly‐prepared nanoensembles **5 a**, **5 b** and **5 c**, electrochemical assessments were performed in dry and N_2_ flushed DMF. Firstly, **ZnPc**, **ZnP** and **BODIPY** derivatives were screened to confirm their intrinsic redox properties and subsequently study their respective electrostatically associated MoS_2_‐based nanoensembles **5 a**, **5 b** and **5 c**. The half wave (E_1/2_) potentials of **ZnPc**,^[44]^ **ZnP** ^[45]^ and **BODIPY** ^[46]^ are in accordance with those reported in literature. Specifically, **ZnPc** exhibits one reversible oxidation, one irreversible and one reversible reduction at +0.09, −1.88 and −1.52 V vs Fc/Fc^+^ respectively (Figure 5). Moving on to the in‐situ formed nanoensemble **5 a**, derived from the titrations already described, we were able to distinguish two reductive waves located at −1.80 and −1.43 V both ~80‐90 mV shifted to more positive potentials, in comparison with the reduction potentials of intact **ZnPc** (Figure 5).

The shift observed is a direct hint of the successful electronic communication between **ZnPc** and MoS_2_ within nanoensemble **5 a**. On the contrary, in the same voltammogram, the oxidation wave was not visible. This was also the case in the voltammograms of **5 b** and **5 c**, where the expected redox waves corresponding to the redox characteristics of **ZnP** and **BODIPY** respectively, were hardly distinguishable due to the nature of the formed nanoensembles, i. e. dispersibility and loading of chromophore that impede the electrochemical study. Briefly, for **ZnP** two reversible reduction and two reversible oxidation waves are registered at −1.74, −1.56, +0.47, +0.75 V vs Fc/Fc^+^, respectively. However, moving to the respective nanoensemble **5 b**, the observed waves at −1.45 V and +0.38 V are hard to be confidently assigned (Figure S12a). On the other hand, while one reversible oxidation (+0.6 V vs Fc/Fc^+^) and one reversible reduction (−1.68 V vs Fc/Fc^+^) are registered for **BODIPY**, only one reduction is hardly observed for the respective MoS_2_‐based nanoensemble **5 c** (−1.53 V vs Fc/Fc^+^) (Figure S12b). It is notable that 150 mV less is needed for the reduction of **BODIPY** to occur, when electrostatically associated with modified MoS_2_ in **5 c**, verifying the electronic interaction between MoS_2_ and **BODIPY** in the ground state. All in all, electrochemical measurements corroborate the successful Coulombic interaction formed during the titration procedures, as well as the electronic communication in the ground state in nanoensembles **5 a** and **5 c**.

## Conclusions

Ultimately, 2H‐MoS₂ was successfully functionalized with two 1,2‐dithiolane derivatives, designed to bear either a carboxylate group or an amine group, specifically to enable versatile electrostatic associations with three different chromophores custom‐selected to absorb light in the down limit, upper limit and heart of the visible region. Nanoensembles formed through electrostatic interactions between functionalized MoS_2_ with the selected counter‐ionized photoactive derivatives of **ZnPc**, **ZnP**, and **BODIPY** were prepared and fully characterized, using a range of spectroscopic, thermal, electrochemical and imaging techniques. Insights into the photophysical properties of each separate system were obtained, revealing distinct behaviors for each nanoensemble. Notably, photoluminescence assays demonstrated strong interactions in the excited state across all photoactive species, while ground‐state interactions exhibited more variable behaviors, indicating a complex interplay dependent on the specific dye‐MoS_2_ system. It is rather interesting that while **ZnPc** and **BODIPY** interact with MoS_2_ in the ground state, as evidenced by UV‐Vis spectroscopy, each follow a different pathway to opposite directions as far as the absorption maximum shift is concerned, namely bathochromic shift for **ZnPc** electrostatically accommodated on MoS_2_, while hypsochromic shift for **BODIPY** electrostatically interacted with MoS_2_. **ZnP** on the other hand, does not exhibit any significant electronic interaction with MoS_2_ in the ground state. A different scenery is set in the emission studies, where all three showcase a strongly quenched emission upon successful formation of the respective nanoensembles, reflecting a charge or energy transfer mechanism between MoS_2_ and each of the chromophores. In addition, in the case of **ZnPc** and **BODIPY**, apart from the strong quenching, a shift of the emission maxima is observed in agreement with the UV‐Vis studies ‐and to opposite directions between them‐ complementary suggesting that custom‐tuning of the optical properties of the final system is possible, based on the specific chromophore employed. As direct consequence, the studied nanoensembles constitute excellent candidates for light harvesting applications, while the electrostatic means of modification described, ensure not only reliable but also potentially reversible interactions. On top of that, the study reveals the influence of the Coulombic association between MoS_2_ and the photoactive molecules on their electronic communication, which is distinctly variable in an analogous covalent and non‐covalent bridging scenario.

## Conflict of Interests

The authors declare no conflict of interest.

1

## Supporting information

As a service to our authors and readers, this journal provides supporting information supplied by the authors. Such materials are peer reviewed and may be re‐organized for online delivery, but are not copy‐edited or typeset. Technical support issues arising from supporting information (other than missing files) should be addressed to the authors.

Supporting Information

## Data Availability

The data that support the findings of this study are available from the corresponding author upon reasonable request.
