# Supplementary material for: Electronic Interactions in Coulombic Associated Photoactive Macrocycles to Chemically Modified MoS2 Nanosheets
Source: Chemistry. 2025 Feb 19;31(19):e202404746. doi: 10.1002/chem.202404746 (PMC11962343; doi:10.1002/chem.202404746)
Supplement: Supplementary file 1 — Supporting Information [file CHEM-31-e202404746-s001.pdf]

Chemistry–A European Journal

Supporting Information

# Electronic Interactions in Coulombic Associated Photoactive Macrocycles to Chemically Modified $MoS_2$ Nanosheets

Marina Tsigkou, Eleni Nikoli, Ioanna K. Sideri, Michalis Kardaras, Hiram Joazet Ojeda Galvan, Mildred Quintana, and Nikos Tagmatarchis*

# SUPPORTING INFORMATION

# Electronic Interactions in Coulombic Associated Photoactive Macrocycles to Chemically Modified $MoS_2$ Nanosheets

Marina Tsigkou,[a]# Eleni Nikoli,[a]# Ioanna K. Sideri,[a] Michalis Kardaras,[a] Hiram Joazet Ojeda Galvan,[b] Mildred Quintana,[b] and Nikos Tagmatarchis*[a]

[a] *Theoretical and Physical Chemistry Institute, National Hellenic Research Foundation, 48 Vassileos Constantinou Avenue, 11635 Athens, Greece.*

[b] *High Resolution Microscopy-CICSaB and Faculty of Science, Universidad, Autonóma de San Luis Potosi, 550 Av. Sierra Leona, Lomas de San Luis Potosi, 78210 SLP, Mexico.*

# *Equal contribution*

## 1. Instrumentation

*$^1$H-NMR spectra* were acquired on a Varian 300 MHz NMR spectrometer at ambient temperature and were internally referenced to residual solvent signals. Data for $^1$H-NMR are reported as follows: chemical shift ($\delta$ ppm), multiplicity (s = singlet, br s = broad singlet, d = doublet, dt = doublet of triplets, dd = doublet of doublet, t = triplet, m = multiplet), coupling constant and integration.

*Tip sonication* was performed with a Bandelin Sonoplus Ultrasonic Homogenizer HD 3200 equipped with a flat head probe (VS70T), running at 35% of the maximum power (250 W).

*Mid-infrared spectra* in the region 500-4500 $cm^{-1}$ were acquired on a Fourier transform IR spectrometer (Equinox 55 from Bruker Optics) equipped with a single reflection diamond ATR accessory (DuraSamp1IR II by SensIR Technologies). Typically, 100 scans were acquired at 2 $cm^{-1}$ resolution.

*Micro-Raman scattering* measurements were performed at room temperature in the backscattering geometry using a RENISHAW in Via Raman spectrometer equipped with a CCD camera and a Leica microscope. As an excitation source, He/Ne lasers (633 nm) was used. A 2400 $mm^{-1}$ line grating for 633 nm laser line was used, providing a spectral resolution of ±1 $cm^{-1}$. Measurements were taken with 10 seconds of exposure times and laser power of ~0.3 mW $cm^{-2}$ to prevent overheating and damage of the basal plane. The laser spot was focused on the sample surface using a long working distance 50× (L50) objective. Raman spectra were collected on numerous spots on the sample and recorded with a Peltier cooled CCD camera. The data were collected and analyzed with Renishaw Wire and Origin software. The intensity ratio $I_{A1g}/I_{2LAM}$ was obtained by taking the peak intensities following any baseline corrections. For the mapping recordings, 5-10 areas of 121 acquisition points each were scanned for every sample and we present here a representative one close to the total average with respect to the intensity ratio $I_{A1g}/I_{2LAM}$. The data were collected and analyzed with Renishaw Wire and Origin software. Raman spectra upon excitation at 633 nm are normalized at the $A_{1g}$ mode for clarity purposes.

*Thermogravimetric analysis* was performed using a TGA Q500 V20.2 Build 27 instrument by TA in an inert atmosphere of nitrogen (purity >99.999%). In a typical experiment, 2 mg of the material was placed in the platinum pan and the temperature was equilibrated at 40 °C. Subsequently, the temperature was increased to 900 °C with a rate of 10 °C/min and the mass changes were recorded as a function of temperature.

*UV-Vis absorption spectra* were recorded on a PerkinElmer (Lambda 19) UV-Vis-NIR spectrophotometer.

*Steady-state emission spectra* were recorded on a Fluorolog-3 JobinYvon-Spex spectrofluorometer (model GL3-21).

*Dynamic light scattering* measurements were performed on an ALV/CGS-3 Compact Goniometer System (ALV GmbH, Germany), equipped with a JDS Uniphase 22 mW He-Ne laser, operating at 632.8 nm, interfaced with an ALV-5000/EPP multi-tau digital correlator with 288 channels and an ALV/LSE-5003 light scattering electronics unit for stepper motor-drive and limit-switch control. The scattering intensity and correlation functions were measured at 90°. Correlation functions were collected ten times and were analyzed using the cumulant method and the CONTIN software, which provide the apparent hydrodynamic radii distributions by Laplace inversion of the correlation function and by aid of the Stokes-Einstein relationship.

*Cyclic voltammetry* was performed on an Autolab PGSTAT128 N potentiostat/galvanostat equipped with a dual mode bipotentiostat (BA module) electrochemical analyzer using a three-electrode system. A platinum button electrode was used as the working electrode. A platinum cloth served as the counter electrode and a platinum wire was used as the reference electrode. Ferrocene/ferrocenium redox couple was used as an internal standard. All solutions were purged prior to electrochemical and spectral measurements using nitrogen gas.

*Transmission electron microscopy* imaging analyses were carried out on a JEM-JEOL-2100 microscope operated at an accelerated voltage of 200 kV. Micrographs were recorded with a 1024 × 1024-pixel, 4K resolution Gatan CCD digital camera. To perform these TEM studies, the sample was dispersed in ethanol and the suspension was ultrasonicated and drop-casted onto a copper carbon holey grid. The element distribution was detected with an Energy Dispersive Spectrometer (EDAX Elite, USA).

## 2. Materials

All solvents and reagents were purchased from Sigma-Aldrich and used without further purification unless stated otherwise.

## 3. Experimental

### Preparation of exfoliated 2H-$MoS_2$

Bulk $MoS_2$ (5.00 g) was dispersed in *N*-Methyl-2-pyrrolidone (500 mL, 99% purity) and placed in an ice bath to undergo tip-sonication (40% amplitude, 5 seconds on- 5 seconds off pulse) for

3 hours. Following ultrasonication, the dispersion was filtered through a polytetrafluoroethylene (PTFE) membrane filter (pore size of 0.2 μm). The resulting grey solid was then redispersed in *N,N*-dimethylformamide (DMF) (300 mL). The dispersion was subsequently centrifuged at 1000 rpm for 15 minutes, and a final filtration was conducted using a PTFE membrane filter (pore size of 0.2 μm) to obtain exfoliated 2H-$MoS_2$ nanosheets (240 mg). UV-Vis spectroscopy (**Figure S1**) confirmed the successful exfoliation, as evidenced by a shift in the exciton peak from 700 nm to 683 nm, indicating an average amount of 15 layers. This number has been calculated using the following equation: $\mathbf{N = 2.3 \times 10^{36}\ e^{-54.888/\lambda_A}}$, where $\lambda_A$ refers to the wavelength of the maximum of absorbance intensity of the A exciton band in the UV-Vis spectrum.[1,2]

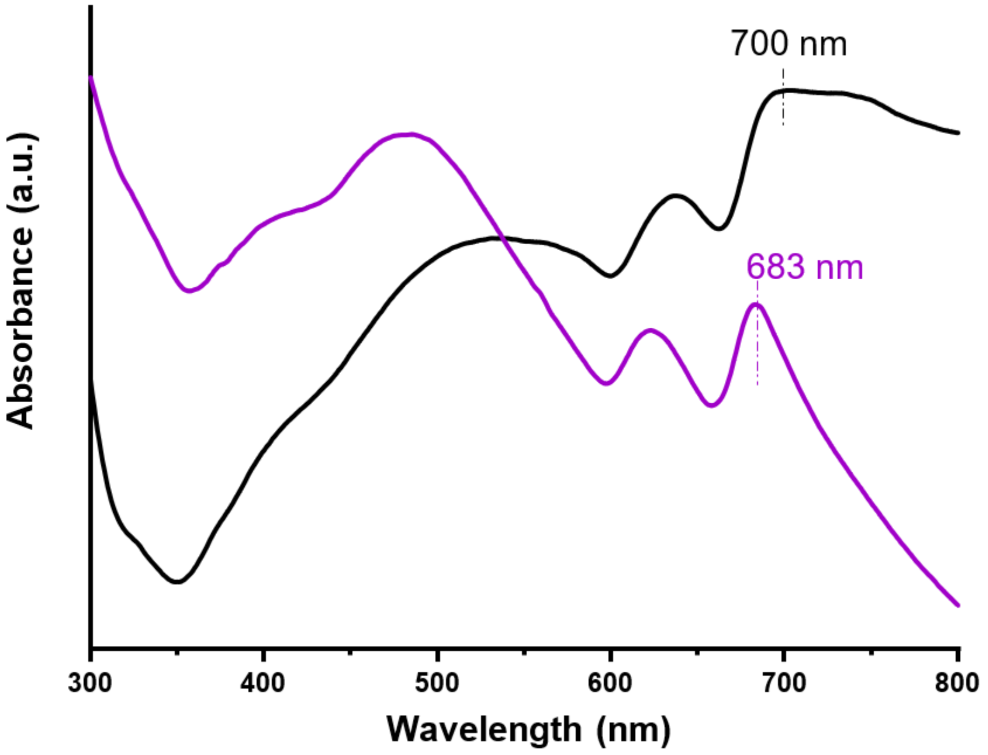


**Figure S1**. UV-Vis spectrum of bulk $MoS_2$ (black) and exfoliated $MoS_2$ (purple) in DMF.

**Synthesis of N-(2-aminoethyl)-S-(1,2-dithiolan-3-yl)pentanamide (2b)**

In a 25 mL two-necked round-bottom flask, lipoic acid (0.5 g, 2.4 mmol) was added and dissolved in dry chloroform (10 mL), followed by the addition of EDCI (1.28 g, 7.28 mmol) and DMAP (0.888 g, 7.28 mmol). The resulting solution was stirred for 30 min under $N_2$ atmosphere and subsequently added dropwise into the solution of ethylenediamine (1.56 mL, 24 mmol) in dry chloroform (20 mL) under $N_2$ which was already set in an ice-bath. The

reaction was kept overnight at room temperature. The next day, the crude mixture was washed with brine and NaOH aqueous solution (1 M) three times. The combined organic phase was dried by anhydrous $Na_2SO_4$. Removal of the organic solvent gives **2b** as a yellow oil (0.2 g, 33% yield). All data are in agreement with those reported in literature.[3] $^1$H-NMR (300 MHz, $CDCl_3$, ppm): $\delta$ 6.22 (s, 1H), 3.40-3.28 (m, 3H), 3.22-3.10 (m, 2H), 2.77 (m, 2H), 2.49-2.43 (m, 1H), 2.37-2.27 (m, 2H), 1.99 (m, 1H), 1.69-1.60 (m, 4H), 1.52-1.43 (m, 3H), 1.19-1.15 (m, 2H).

**Preparation of modified materials (3a) and (3b)**

For the preparation of **3a**, the 1,2-dithiolane derivative **2a** (20 mg) and exfoliated 2H-$MoS_2$ nanosheets **1** (40 mg) were dispersed in DMF (15 mL). Similarly, for the synthesis of material **3b**, derivative **2b** (200 mg) and exfoliated 2H-$MoS_2$ **1** (80 mg) were dispersed in DMF (20 mL). Both dispersions were stirred at 70 °C for 36 hours. Subsequently, the mixtures were filtered using a polytetrafluoroethylene (PTFE) membrane filter (pore size of 0.2 μm), and thoroughly washed with copious amounts of DMF, MeOH and DCM to ensure complete removal of any non-covalently attached organic moieties.

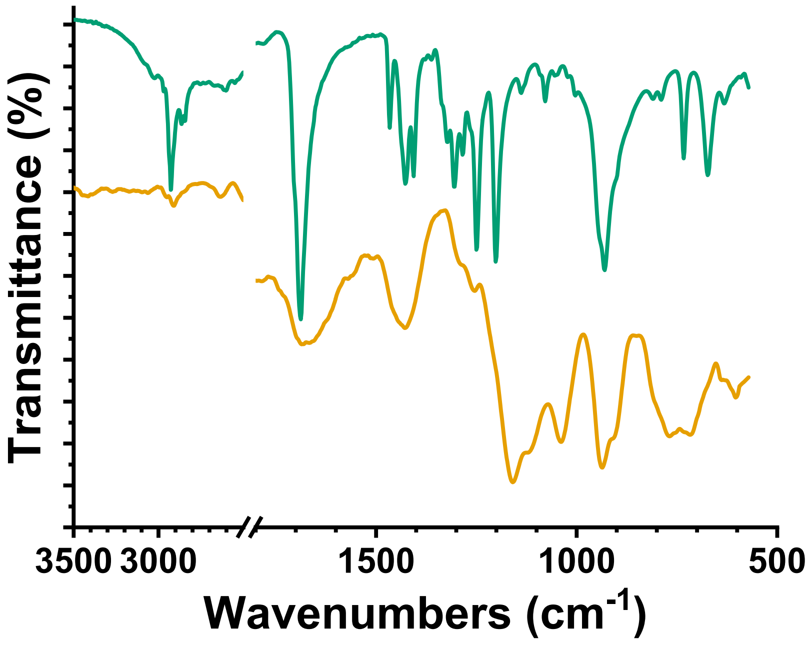


**Figure S2.** FT-IR spectra of α-lipoic acid **2a** (green), and modified material **3a** (orange).

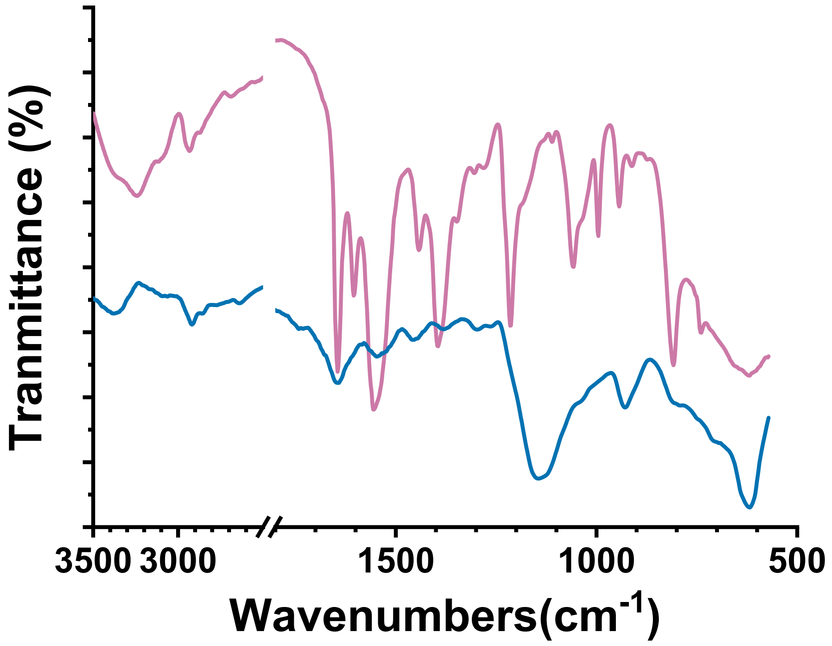


**Figure S3.** FT-IR spectra of 1,2-dithiolane derivative **2b** (purple) and modified material **3b** (blue).

**Quantification of the functionalization by TGA (degree of functionalization)**[4]

$$\boldsymbol{organic\ chain\ per\ MoS2\ unit} = \frac{(1-\mathrm{X})\ \cdot MW\ (moiety\ attached)}{\mathrm{X}\cdot MW(MoS2)}\cdot \mathbf{100}$$

$$X = weight\ loss, as\ derived\ from\ the\ TGA\ diagram$$

**Titration procedure**

A solution of positively charged ammonium-modified zinc-phthalocyanine (**ZnPc**) was titrated with **4a**, bearing a negative charge. To prepare the **ZnPc** $10^{-6}$ M solution, a stock solution was first prepared by dissolving **ZnPc** (2.3 mg) in DMF (20 mL), where aqueous HCl (0.8 M, 5 µL) was added to achieve ionization (solution A1). From this stock solution A, 10 µL was diluted in 2 mL of DMF to achieve the desired concentration of $10^{-6}$ M (solution B1). For the modified material **4a**, the dispersion was prepared by adding **3a** (5.36 mg) in DMF (20 mL) followed by the addition of aqueous NaOH (0.05 M, 5µL) to deprotonate the carboxylic acid groups. Additionally, 10 µL of the **ZnPc** stock solution was added to ensure a constant **ZnPc** concentration ($10^{-6}$ M) throughout the titration (solution C1). In 2.5 mL of solution B1, increment additions of solution C were performed. The following values represent the final volume of **4a** (solution C1) in the titration mixture: + 10µL, + 30 µL, + 80 µL, +130 µL, +180

μL, + 230 μL, + 300 μL, +400 μL, +500 μL, +600 μL, +700 μL, +800 μL, + 900 μL, +1mL, +1.1 mL, +1.2 mL, +1.3 mL, +1.4 mL, +1.5 mL, +1.7 mL.

Following the same procedure, a solution of zinc-porphyrin (**ZnP)** was titrated using material **4b**. To prepare the **ZnP** solution, a stock solution was first made by dissolving zinc-porphyrin (3.61 mg) in DMF (25mL) followed by the addition of NaOH (0.5 M, 10μL) (solution A2). From this stock solution, 10 μL was then diluted in 2 mL of DMF to achieve the desired concentration of $10^{-6}$ M (solution B2). For the modified material **4b**, the dispersion was prepared by adding **3b** (1.08 mg) in DMF (5 mL) followed by the addition of aqueous HCl (0.001 M, 1.6 μL) and 25 μL of the **ZnP** stock solution (solution B2) to ensure a constant **ZnP** concentration ($10^{-6}$ M) throughout the titration procedure. In 2.5 mL of **ZnP** solution B2, increment additions of solution C2 were performed. The following values represent the final volume of **4b** (solution C2) in the titration mixture: + 10μL, + 20 μL, + 40 μL, +80 μL, +160 μL, + 480 μL, + 960 μL, + 1.92 mL, + 3 mL, + 5 mL, + 10 mL, +16.5 mL.

Finally, a solution of **BODIPY** was titrated using material **4b**. To prepare the **BODIPY** solution, a stock solution was first made by dissolving **BODIPY** (2.31 mg) in DMF (25 mL) followed by the addition of aqueous NaOH (0.5 M, 10 μL) (solution A3). From this stock solution, 10 μL was then diluted in 2 mL of DMF to achieve the desired concentration of $10^{-6}$ M (solution B3). For the modified material **4b**, the dispersion was prepared by adding **3b** (4.45 mg) in DMF (20 mL) followed by the addition of aqueous HCl (0.01 M, 7 μL) and 100 μL of the **BODIPY** stock solution was added to ensure a constant **BODIPY** concentration ($10^{-6}$ M) throughout the titration procedure. In 2.5 mL of **BODIPY** solution B3, increment additions of solution C3 were performed. The following values represent the final volume of (**4b**) (solution C3) in the titration mixture: + 10μL, + 80 μL, + 200 μL, + 500 μL, + 1 mL, + 2 mL, +3.5 mL, + 6 mL, + 9.5 mL.

Each addition was followed by UV-Vis spectroscopy and photoluminescence (PL) spectroscopy measurements. The resulting electrostatically associated $MoS_2$/chromophore nanoensembles **5a**, **5b**, and **5c** were studied by employing absorption and emission spectroscopic methods to unveil the electronic interactions taking place between the different counterparts in each system.

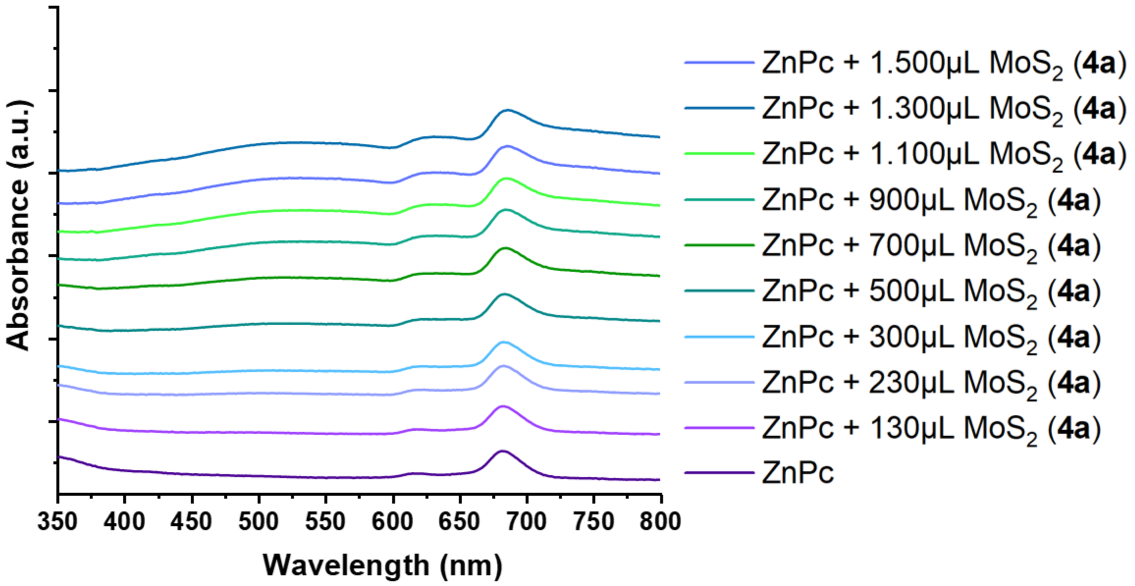


**Figure S4.** Absorption spectra of **ZnPc** upon sequential additions of **4a** in DMF.

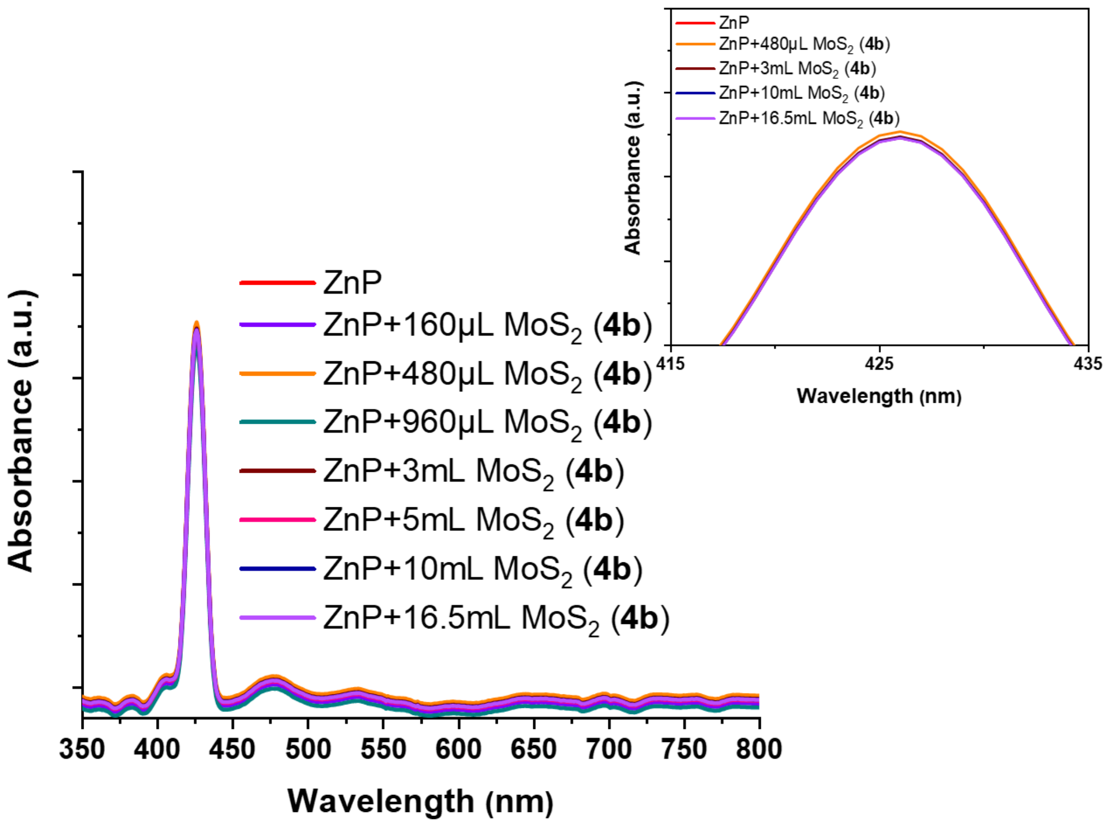


**Figure S5**. Absorption spectra of **ZnP** upon sequential additions of **4b** in DMF**,** after subtraction of the $MoS_2$ absorption background**. Inset**: magnified view of the spectral region between 415 and 435 nm.

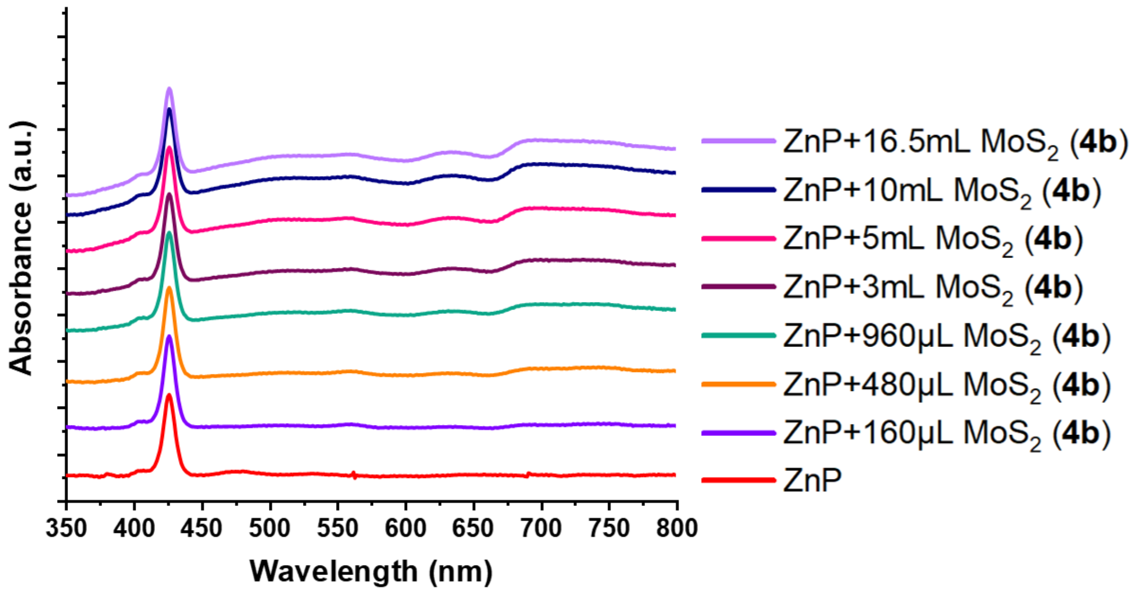


**Figure S6.** Absorption spectra of **ZnP** upon sequential additions of **4b** in DMF.

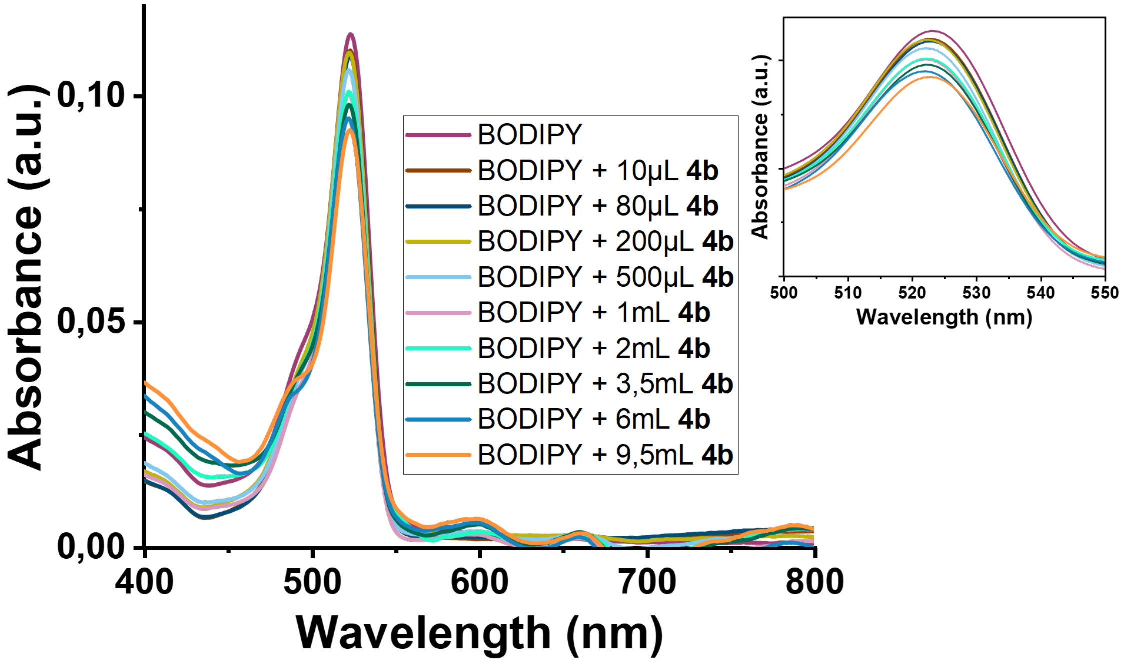


**Figure S7.** Absorption spectra of **BODIPY** upon sequential additions of **4b** in DMF, after subtraction of the $MoS_2$ absorption background. **Inset**: magnified view of the spectral region between 515 and 535 nm.

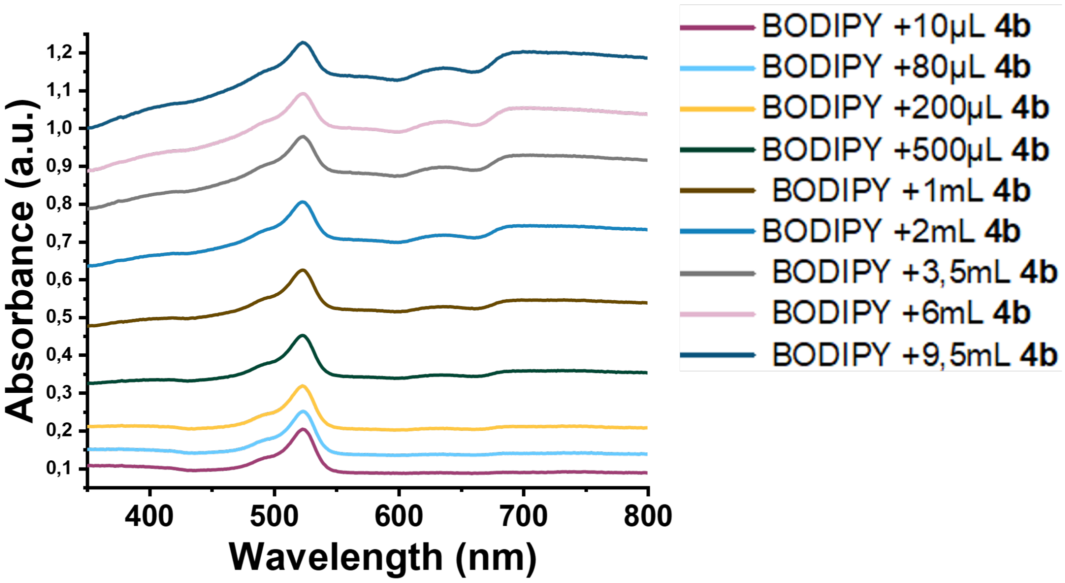


**Figure S8.** Absorption spectra of **BODIPY** upon sequential additions of **4b** in DMF.

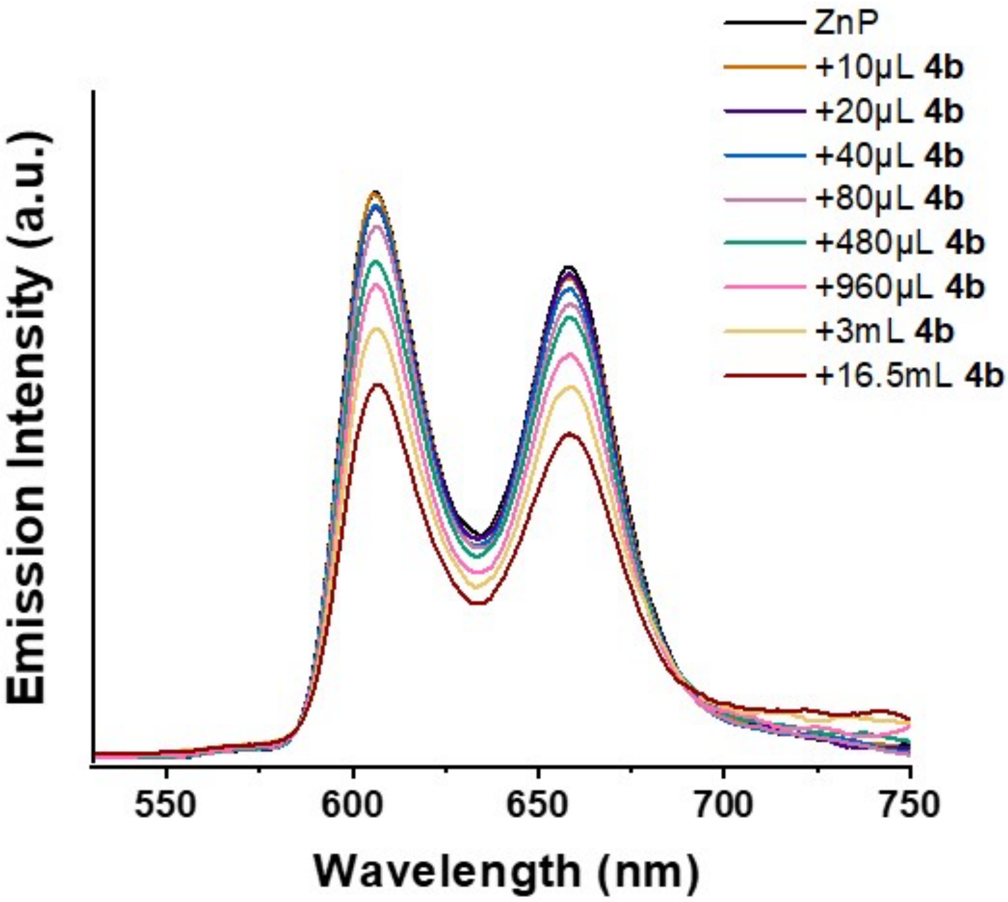


**Figure S9.** Emission spectra of **ZnP** upon incremental additions of **4b** in DMF ($\lambda_{exc}$ 462 nm).

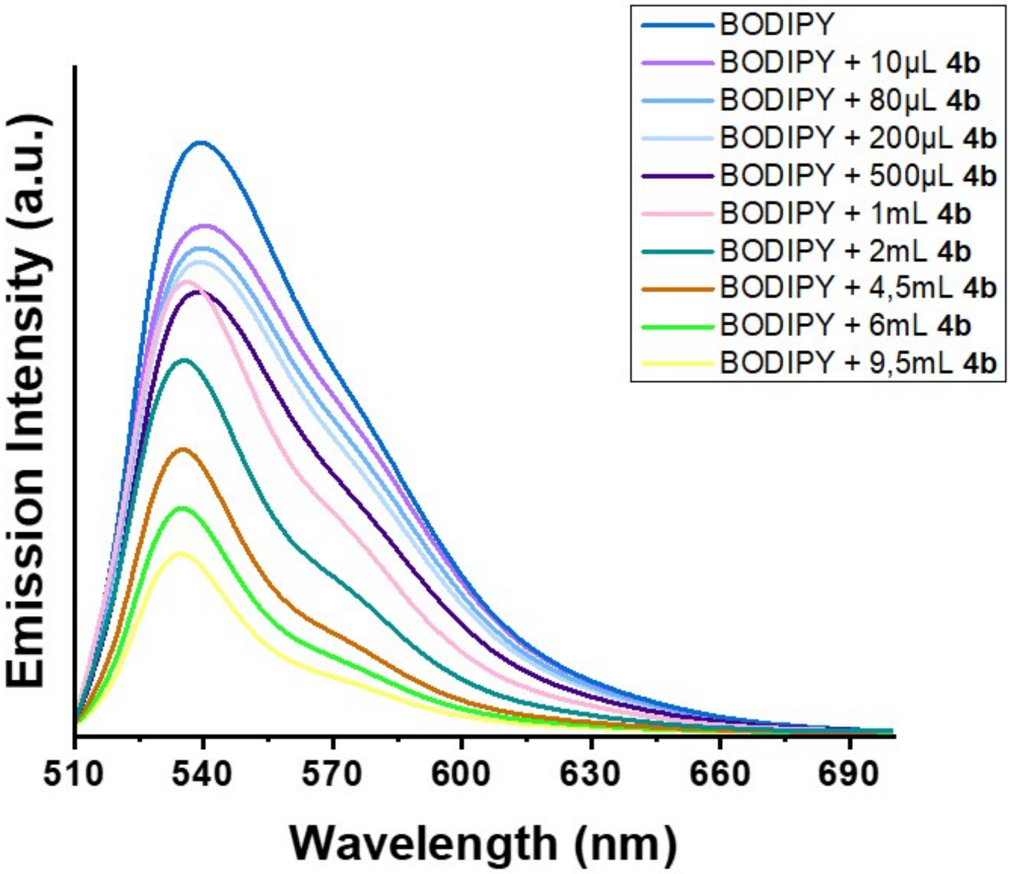


**Figure S10.** Emission spectra of **BODIPY** upon incremental additions of **4b** in DMF ($\lambda_{exc}$ 490 nm).

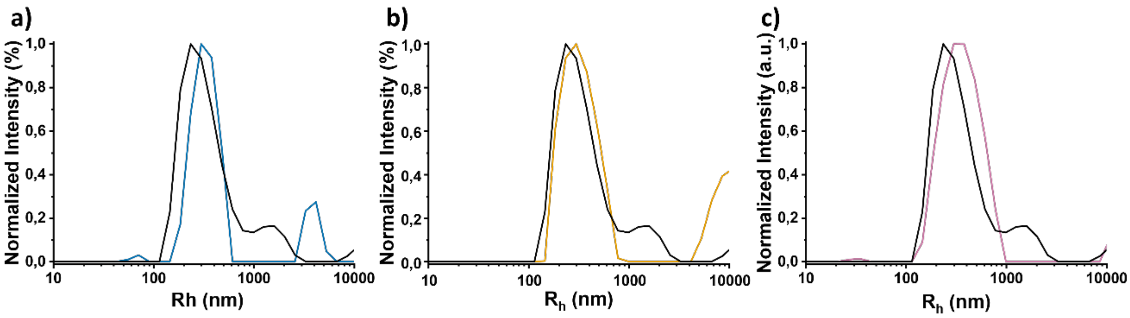


**Figure S11.** Dynamic light scattering measurements in DMF in 90° of nanoensemble **a) 5a** (blue), **b) 5b** (yellow), and **c) 5c** (pink), in comparison with exfoliated $MoS_2$ nanosheets **1** (black).

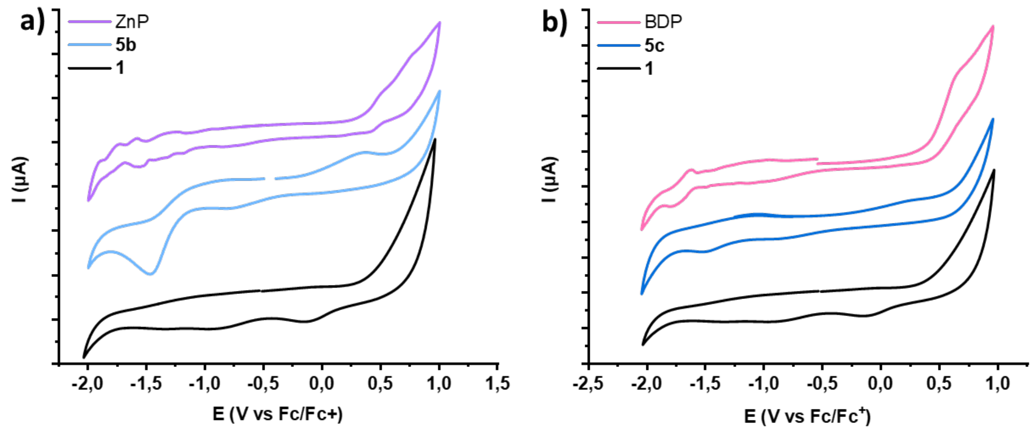


**Figure S12.** Cyclic voltammograms **a)** of exfoliated $MoS_2$ nanosheets **1**, **ZnP** and nanoensemble **5b**, **b)** of exfoliated $MoS_2$ nanosheets **1**, **BODIPY** and nanoensemble **5c**, in nitrogen saturated 0.1 M $TBAPF_6$ in dry DMF.

**References**

1. C. Backes, D. Campi, B. M. Szydlowska, K. Synnatschke, E. Ojala, F. Rashvand, A. Harvey, A. Griffin, Z. Sofer, N. Marzari, J. N. Coleman, D. D. O'Regan, *ACS Nano* **2019**, *13*, 7050.
2. C. Backes, R. J. Smith, N. McEvoy, N. C. Berner, D. McCloskey, H. C. Nerl, A. O'Neill, P. J. King, T. Higgins, D. Hanlon, N. Scheuschner, J. Maultzsch, L. Houben, G. S. Duesberg, J. F. Donegan, V. Nicolosi, J. N. Coleman, *Nat. Commun*. **2014**, *5*, 4576.
3. M. Sun, L. Yang, P. Jose, L. Wang, J. Zweit, *J. Mater. Chem. B* **2013**, *1*, 6137.
4. R. Quirós-Ovies, M. V. Sulleiro, M. Vera-Hidalgo, J. Prieto, I. J. Gómez, V. Sebastián, J. Santamaría, E. M. Pérez, *Chem. Eur. J.* **2020**, *26*, 6629.
